# Supplementary material for: MWCNT Decorated Rich N-Doped Porous Carbon with Tunable Porosity for CO2 Capture
Source: Molecules. 2021 Jun 7;26(11):3451. doi: 10.3390/molecules26113451 (PMC8201232; doi:10.3390/molecules26113451)
Supplement: Supplementary file 1 [file molecules-26-03451-s001.zip › molecules-1222512-SI.pdf]

Supporting Information

CO_2 absorption experiment. CO_2 adsorption capacity was measured by a self-made static adsorption apparatus as illustrated in the scheme below. The apparatus mainly includes gas cylinders (CO_2, N_2, and He), constant temperature adsorption cell, vacuum pump, and pressure sensing device. The vacuum pump provided a vacuum environment for the whole apparatus, and the adsorption device was placed in a water bath to ensure the constant temperature adsorption. The pressure of the experimental system was measured by a pressure sensing device.

In this apparatus, a certain mass of adsorbents was loaded into the adsorption cell, then the whole apparatus was vacuumed. Next, CO_2 with a certain pressure was pumped into the pressure cell. Finally opening the adsorption cell valve so that the gas pressure in the two cells can achieve equilibrium. Through the available volume of the two cells and the inside pressure data, the molar volume of gas can be calculated by thermodynamic equation to obtain the adsorption capacity of the material.

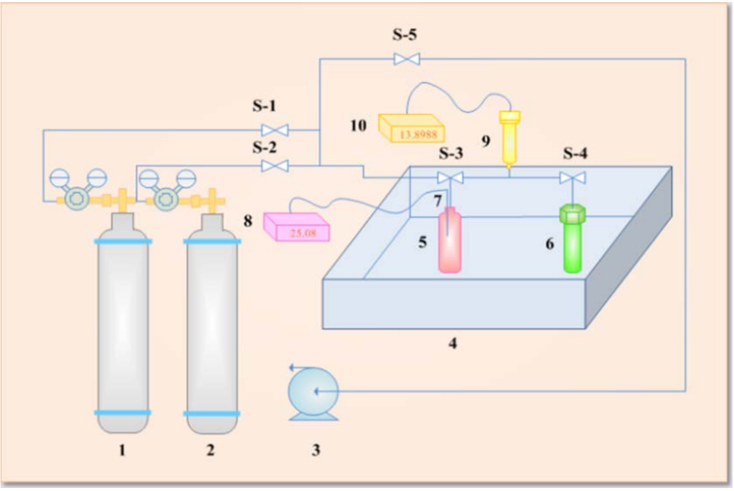

Scheme S1. Diagram of the experimental apparatus: (1) He steel cylinder, (2) CO_2 steel cylinder, (3) Vacuum pump, (4) Thermostat water bath, (5) Reactor vessel, (6) Adsorption vessel, (7) Temperature controller, (8) Temperature monitors, (9) Pressure transducer, (10) Pressure monitors.

The Redlich Kwong (RK) equation was used to calculate the molar volume of gas (Eq. S1). The environmental parameters, the degree of vacuum pumping and other factors led to the difference in the effective volume of the adsorption cell. Helium gas was pumped into the apparatus which was not adsorbed by adsorbents. Eq. S2 was used to correct the effective volume of the adsorption cell. Eq. S3 was used to calculate the CO_2 adsorption capacity at the target pressure. Eq. S4 was used to calculate the accumulated CO_2 adsorption capacity at multiple target pressures, which was applied to test adsorption isotherm curves.

$$P = \frac{RT}{V-b} - \frac{a}{T^{1/2}V(V+b)} \quad \text{(S1)}$$

$$V_{fs} = \frac{V_p(v_{He\text{-}pfs} - v_{He\text{-}p})}{v_{He\text{-}p}} \quad \text{(S2)}$$

$$n_i = \frac{V_p}{v_{p,i}m} - \frac{V_p+V_{fs}}{v_{pfs,i}m} \qquad i=1 \quad \text{(S3)}$$

$$n_i = n_{i\text{-}1} + \frac{V_p}{v_{p,i}m} + \frac{V_{fs}}{v_{pfs,i\text{-}1}m} - \frac{V_p+V_{fs}}{v_{pfs,i}m} \qquad i=2,3,4\ldots\ldots \quad \text{(S4)}$$

where V_p (mL) is pressure cell volume; V_{fs} (mL) is effective volume of adsorption cell; $v_{He\text{-}p}$ (mL/mmol) is molar volume of helium filled with pressure cell; $v_{He\text{-}pfs}$ (mL/mmol) is molar volume of helium gas when adsorption equilibrium is reached; $v_{p,i}$ (mL/mmol) is molar volume of CO_2 in pressure cell; $v_{pfs,i}$ (mL/mmol) is molar volume of CO_2 when adsorption equilibrium is reached; n_i (mmol/g) is CO_2 adsorption capacity at target pressures; m (g) is mass of adsorbent; and i is number of times to achieve equilibrium.

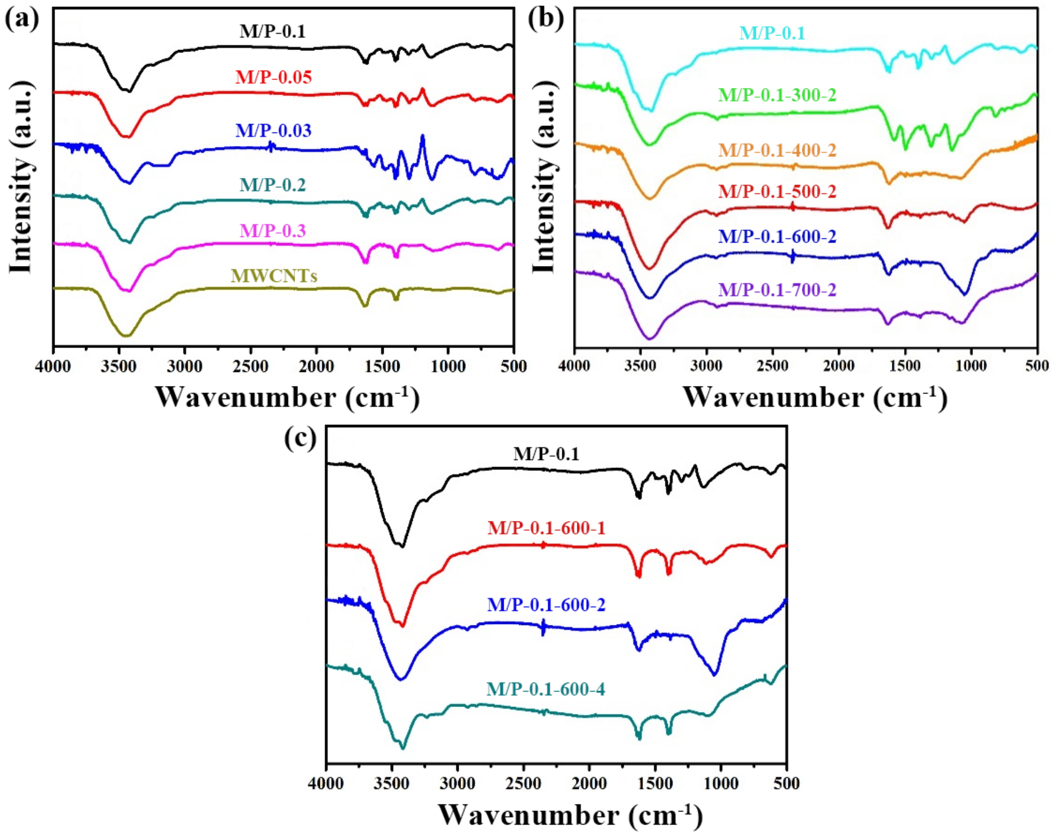

Figure S1. FTIR spectra of (a) M/P-x (x = 0, 0.03, 0.05, 0.1, 0.2, and 0.3), (b) M/P-0.1-T-2 (T = 0, 300, 400, 500, 600, and 700), and (c) M/P-0.1-600-y (y = 0, 1, 2, and 4).

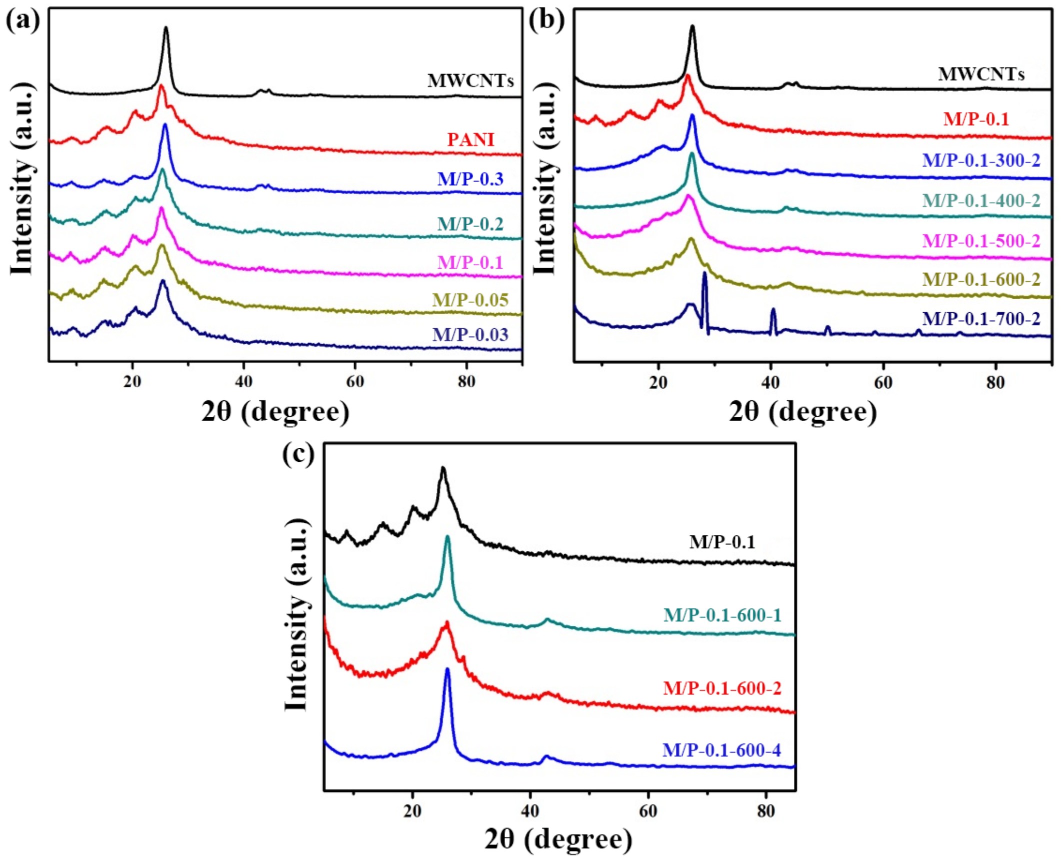

Figure S2. The XRD patterns of the precursor (a) M/P-x (x = 0, 0.03, 0.05, 0.1, 0.2, and 0.3), (b) M/P-0.1-T (T = 0, 300, 400, 500, 600, and 700), and (c) M/P-0.1-600-y (y = 0, 1, 2, and 4).

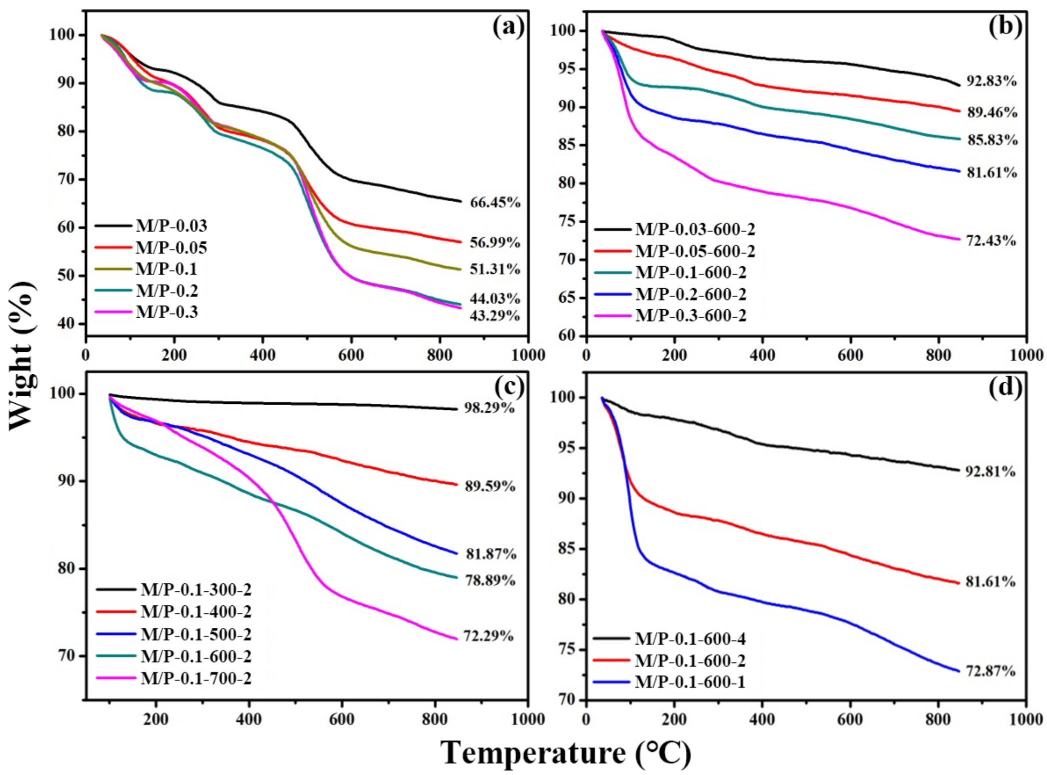

Figure S3. The TG curves of (a) M/P-x (x = 0, 0.03, 0.05, 0.1, 0.2, and 0.3), (b) M/P-x-600-2 (x = 0, 0.03, 0.05, 0.1, 0.2, and 0.3), (c) M/P-0.1-T (T = 0, 300, 400, 500, 600, and 700), and (d) M/P-0.1-600-y (y = 1, 2, and 4).

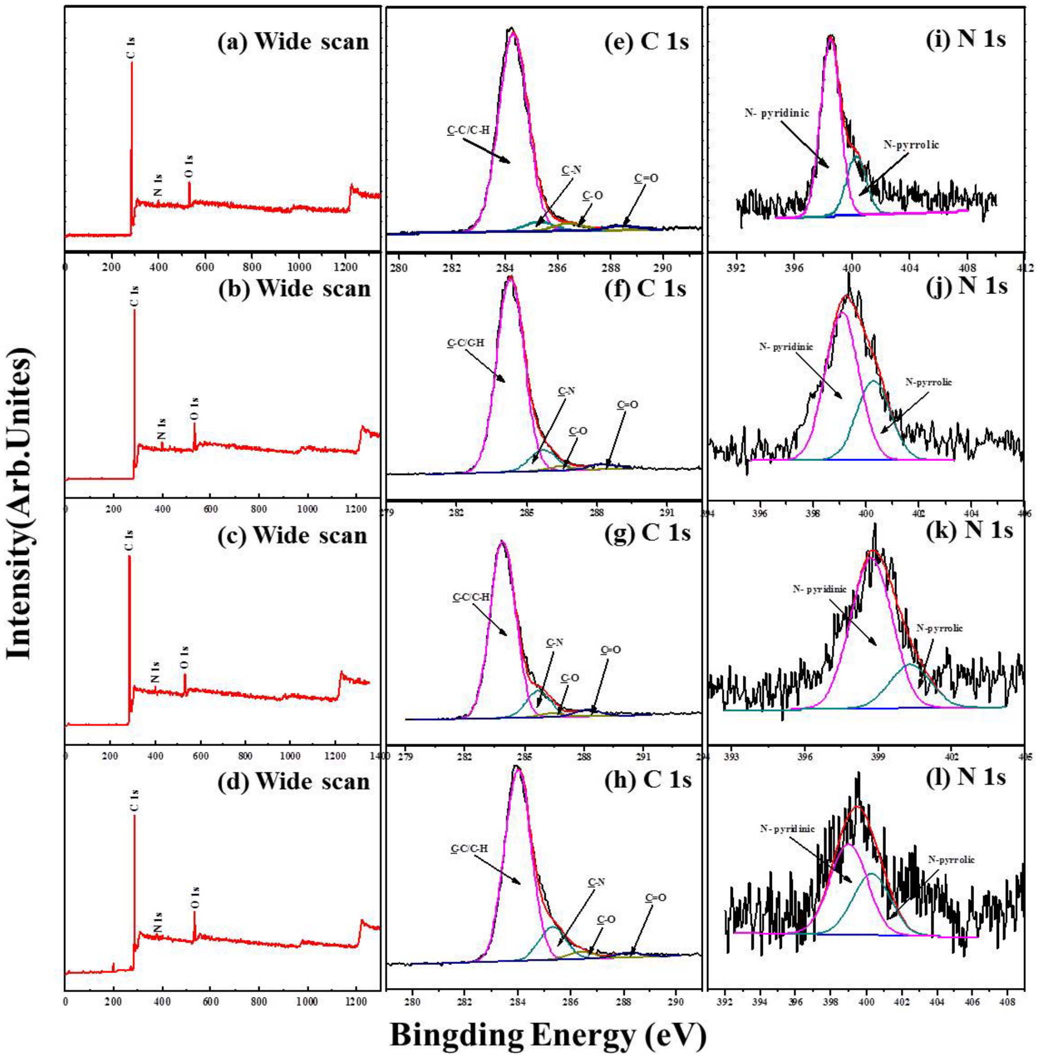

Figure S4. The XPS spectra of M/P-0.1-T-2 (T = 300, 400, 500, and 600). (a) Wide scan, (e) C 1s, and (i) N 1s regions of M/P-0.1-300-2. (b) Wide scan, (f) C 1s, and (j) N 1s regions of M/P-0.1-400-2. (c) Wide scan, (g) C 1s, and (k) N 1s regions of M/P-0.1-500-2. (d) Wide scan, (h) C 1s, and (l) N 1s regions of M/P-0.1-600-2.

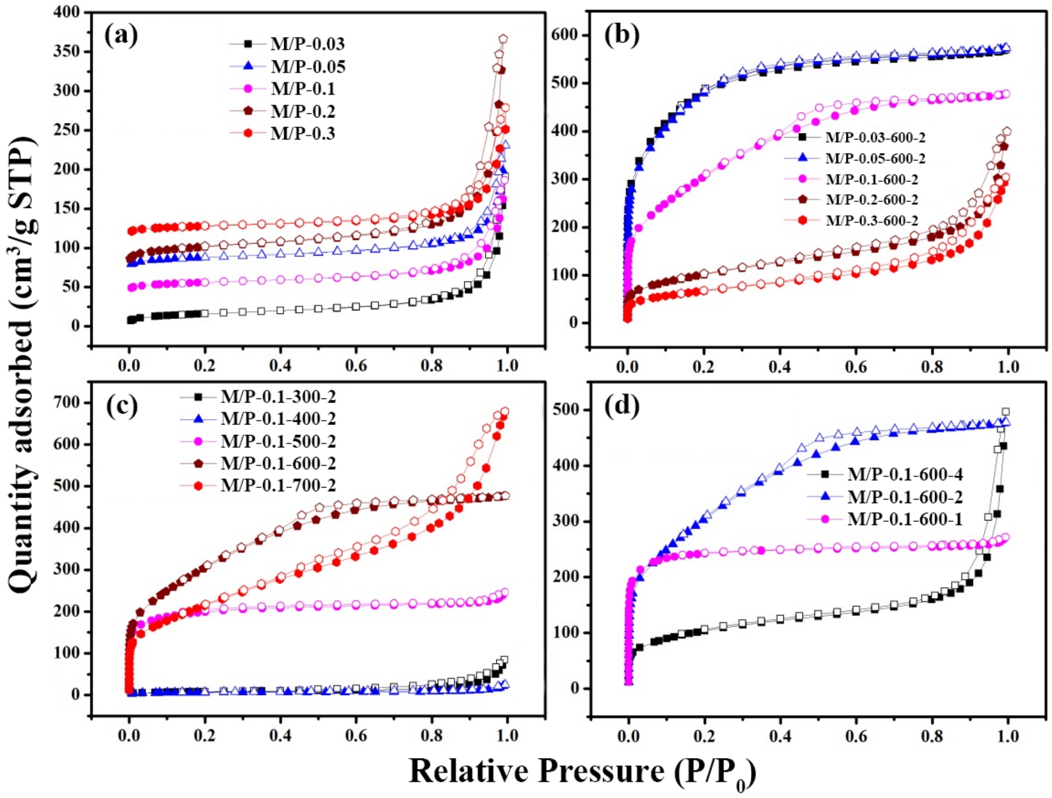

Figure S5. N_2 adsorption-desorption isotherms of (a) M/P-x (x = 0, 0.03, 0.05, 0.1, 0.2, and 0.3), (b) M/P-x-600-2 (x = 0, 0.03, 0.05, 0.1, 0.2, and 0.3), (c) M/P-0.1-T (T = 0, 300, 400, 500, 600, and 700), and (d) M/P-0.1-600-y (y = 1, 2, and 4).

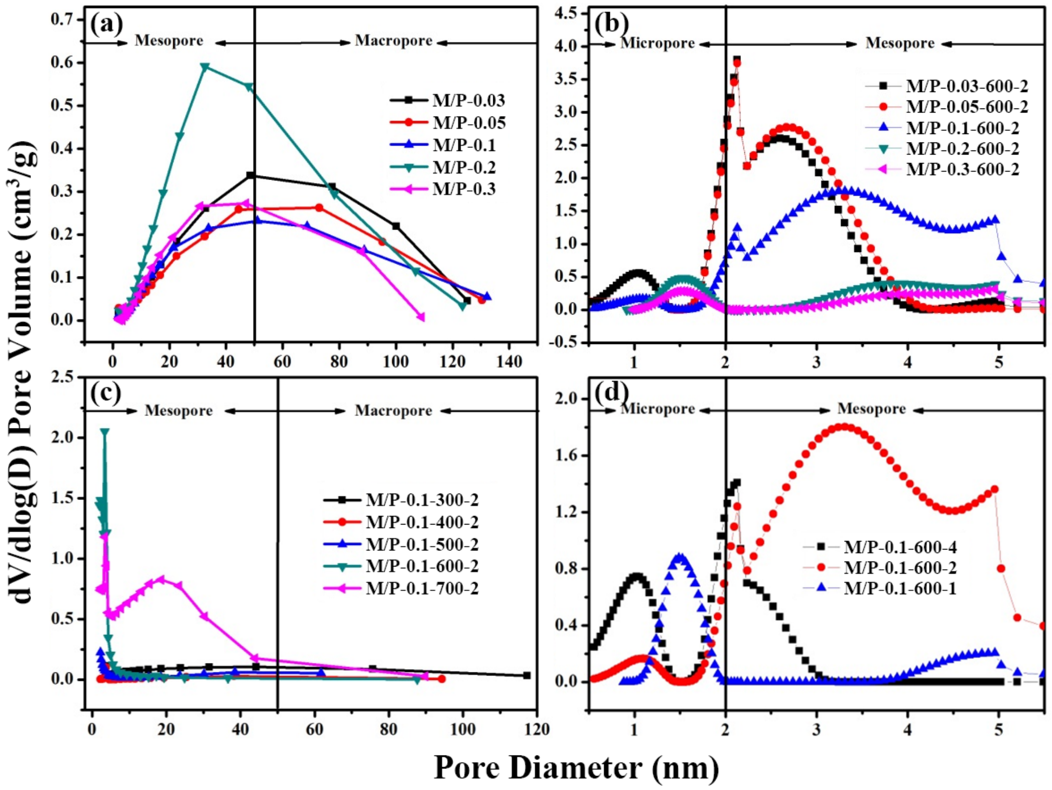

Figure S6. The pore size distribution profiles of (a) M/P-x (x = 0, 0.03, 0.05, 0.1, 0.2, and 0.3), (b) M/P-x-600-2 (x = 0, 0.03, 0.05, 0.1, 0.2, and 0.3), (c) M/P-0.1-T (T = 0, 300, 400, 500, 600, and 700), and (d) M/P-0.1-600-y (y = 1, 2, and 4).

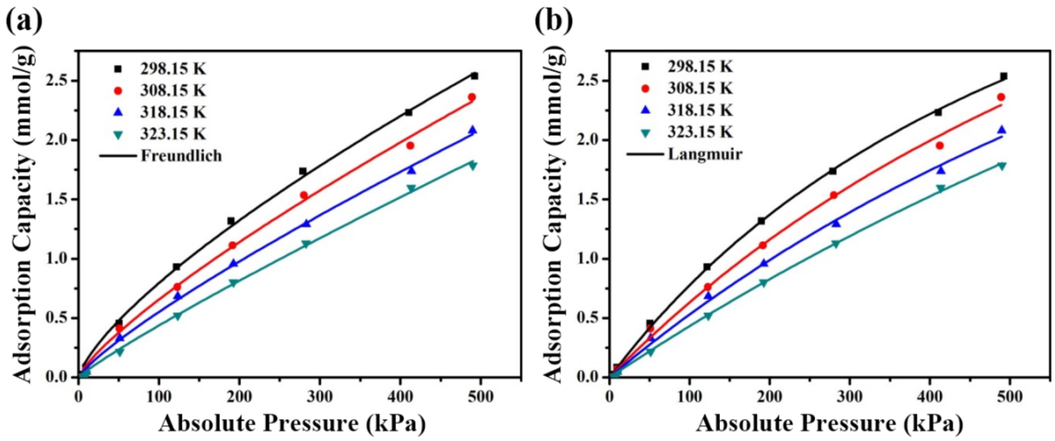

Figure S7. (a) Freundlich and (b) Langmuir fitted adsorption isotherms of M/P-0.1-600-2 at different temperatures.

Table S1. N content of M/P-0.1-T-2 (T = 0, 300, 400, 500, 600, and 700).

Sample	N (wt%)	C (wt%)	O (wt%)
**M/P-0.1**	4.14	89.30	6.56
**M/P-0.1-300-2**	3.10	89.07	7.83
**M/P-0.1-400-2**	3.69	86.40	9.91
**M/P-0.1-500-2**	3.17	89.31	7.52
**M/P-0.1-600-2**	3.11	88.31	8.58
**M/P-0.1-700-2**	2.76	86.34	10.90
**M/P-0.05-600-2**	3.10	86.70	10.20
**M/P-0.03-600-2**	2.91	88.20	8.89

Table S2. BET data and CO_2 adsorption capacity of all samples.

Sample	S_{BET}[a] (m^2/g)	Pore volume[b] (cm^3/g)	Pore size[c] (nm)	CO_2 adsorption capacity[d] (mmol/g)
MWCNTs	104	0.48	19.4	0.18
PANI	42	0.18	27.3	0.54
M/P-0.03	47	0.26	23.9	0.69
M/P-0.05	45	0.21	20.1	0.72
M/P-0.1	58	0.22	23.0	0.83
M/P-0.2	66	0.23	21.5	0.75
M/P-0.3	87	0.44	24.0	0.65
M/P-0.1-300-2	213	0.026	22.9	1.10
M/P-0.1-400-2	289	0.13	10.5	1.19
M/P-0.1-500-2	611	0.18	4.83	1.76
M/P-0.1-600-2	1017	0.37	2.71	2.63
M/P-0.1-700-2	780	0.94	6.42	1.83
M/P-0.1-600-4	355	0.62	14.4	1.62
M/P-0.1-600-1	740	0.062	5.13	1.85
M/P-0.03-600-2	1570	0.24	2.95	1.25
M/P-0.05-600-2	1601	0.27	2.87	1.63
M/P-0.2-600-2	359	0.53	6.78	1.75
M/P-0.3-600-2	238	0.44	9.44	1.46
M/P-0-600-2	-	-	-	1.16

[a] BET surface area. [b]BJH adsorption cumulative volume of pores between 17.000 and 3,000.000 Å width. [c]BJH adsorption average pore width (4V/A). [d] CO_2 adsorption capacity at 298 K, 5 bar.

Table S3. CO_2 adsorption ability of M/P-0.1-600-2 and reported adsorbents in CO_2 capture.

Adsorbents	Temperature (K)	Pressure	CO_2 adsorption capacity(mmol g^{-1})	Ref.
M/P-0.1-600-2	298	5 bar	2.63	This work
Heptazine-based Polymeric porous Framework	273	1 bar	2.35	[1]
Chestnut biomass carbon	298	1 bar	2.14	[2]
Phloroglucinol based Microporous polymeric Organic frameworks	298	1 bar	2.02	[3]
Microporous organic Polymers (CTHP)	298	1bar	1.90	[4]
Amine-grafted SBA-15	298	1 bar	1.88	[5]
Melamine–formaldehyde Resins N-doped carbon	298	1 bar	1.86	[6]
Amine-functionalized MIL-53 (Al) MOFs	298	1 bar	1.82	[7]
Mesoporous Carbon	298	1 bar	1.50	[8]
Amine-grafted Zeolite ITQ-6	293	1 bar	1.21	[9]

Table S4. Model fitting parameters of CO_2 adsorption isotherms on M/P-0.1-600-2 at different temperatures.

Models	Fitted Parameters	Temperature (K)			
		298.15 K	308.15 K	318.15 K	323.15 K
Freundlich	K_F (mmol • g^{-1} • kPa^{-n})	0.02705	0.01679	0.01236	0.00701
	n	1.36174	1.25596	1.21213	1.11421
	χ^2(×10^{-4})	24.7	24.7	12	8.4438
	R^2	0.9973	0.99674	0.99797	0.99823
Langmuir	Q_{max}(mmol • g^{-1})	2.5312	2.3143	1.9324	1.8023
	K_L(kPa^{-1})	0.00154	9.82782E-4	7.78396E-4	4.6644E-4
	χ^2(×10^{-4})	5.738	30.8	19.2	3.411
	R^2	0.99937	0.99593	0.99675	0.99929
Redlich -Peterson	a_R(kPa^{-bR})	0.01023	0.14176	0.30359	2.69341E-9
	K_R(mmol • g^{-1} • kPa^{-n})	0.01041	0.01209	0.01203	0.00417
	b_R	0.74385	0.38689	0.2949	2.87064
	χ^2(×10^{-4})	3.9993	26.9	13.6	1.9832
	R^2	0.99956	0.99645	0.9977	0.99959

K_L and K_F represent Langmuir balanced adsorption constant and Freundlich adsorption constant. K_R, a_R, b_R are empirical constants of Redlich-Peterson isotherm equation.

Table S5. Kinetic parameters of CO_2 adsorption on M/P-0.1-600-2.

**Samples**	**Models**	Fitted Parameters					
		k_F (min^{-n})	k_L (min^{-1})	Q_e (mmol·g^{-1})	n	χ^2(×10^{-4})	R^2
**M/P-0.1-600-2**	Fickian diffusion	1.568	-	2.621	0.0123	3.50	0.999
	LDF	-	5.374	2.564	-	21.30	0.996

References

[1] Dang, QQ.; Zhan, YF.; Wang, XM.; Zhang, XM. *ACS Appl. Mater. Interfaces* **2015**, 7, 28452－28458.

[2] Nelson, KM.; Mahurin, SM.; Mayes, RT.; Williamson, B.; Teague, CM.; Binder, AJ.; Baggetto, L.; Veith GM.; Dai, S. *Micropor. Mesopor. Mat.* **2016**, *222*, 94－103.

[3] Katsoulidis, AP.; Kanatzidis, MG. *Chem. Mater.* **2011**,*23*, 1818－1824.

[4] Gao, H.; Ding, L.; Bai, H.; Li, L. *ChemSusChem* **2017**, *10*, 618–623.

[5] Lashaki, MJ.; Sayari, A. *Chem. Eng. J.* **2018**, *334*, 1260－1269.

[6] Pevida, C; Drage, TC; Snape, CE. *Carbon* **2008**, *46*, 1464－1474.

[7] Kim, J.; Kim, WY.; Ahn, W-S. *Fuel* **2012**, *102*, 574－579.

[8] Peng, X.; Zhang, Q.; Cheng, X.; Cao, D. *Acta phys-Chim. Sin.* **2011**, *27*, 2065–2071.

[9] Zukal, A.; Dominguez, I.; Mayerova, J.; Cejka, J. *Langmuir* **2009**, *25*, 10314－10321.
